# pVHL status in clear cell renal cell carcinoma regulates HIF-α and E-cadherin expression levels and their implications for tumor progression

**DOI:** 10.48101/ujms.v130.12982

**Published:** 2025-12-22

**Authors:** Raviprakash T. Sitaram, Börje Ljungberg

**Affiliations:** a Department of Odontology, Umeå University, Umeå, Sweden; b Department of Diagnostics and Intervention, Urology and Andrology, Umeå University, Umeå, Sweden

**Keywords:** Renal cell carcinoma, ccRCC, E-cadherin, HIF-1α, HIF-2α, HIF-3α, EMT, tumor progression

## Abstract

**Objectives:**

This study aimed to determine the effects of von Hippel–Lindau protein (VHL) expression on hypoxia-inducible factor (HIF) and E-cadherin proteins. Furthermore, to evaluate the influence of the VHL–HIF–E-cadherin pathway in clear cell renal cell carcinoma (ccRCC).

**Materials and Methods:**

This study used tissue samples collected from 150 patients with ccRCC and 24 adjacent kidney cortex samples. Immunoblotting was performed to measure the expression levels of VHL and E-cadherin. Additionally, nuclear expression of HIF-α was evaluated by immunohistochemistry (IHC) using a tissue microarray (TMA).

**Results:**

pVHL levels were lower in ccRCC than in the adjacent kidney cortex; however, pVHL levels showed no correlation with clinicopathological parameters. Nuclear HIF-1α levels were higher in stage IV tumors, whereas HIF-2α levels increased with tumor size. No correlation was observed between HIF-3α levels and clinicopathological parameters. E-cadherin protein expression was reduced in ccRCC tissues and in higher-stage and larger tumors. In pVHL-high ccRCC, E-cadherin levels were lower in advanced-stage and larger tumors. Higher levels of HIF-1α and HIF-3α were observed in pVHL-low tumors. E-cadherin expression negatively correlated with nuclear HIF-1α expression. In pVHL-high ccRCCs, E-cadherin was negatively correlated with HIF-1α, while in pVHL-low ccRCCs, E-cadherin was negatively correlated with HIF-2α. E-cadherin was not associated with cancer-specific survival in patients with pVHL-low tumors, whereas E-cadherin expression was linked to improved survival in patients with pVHL-high tumors.

**Conclusion:**

VHL inactivation causes HIF-α activation and suppresses E-cadherin expression, thereby promoting ccRCC progression. This study provides insights into the potential biomarkers and therapeutic targets for ccRCC treatment.

## Introduction

Clear cell renal cell carcinoma (ccRCC), the most prevalent RCC type, comprises approximately 70–80% of all RCCs (1, 2). Clear cell RCC is an aggressive cancer that originates from the epithelial cells of the proximal convoluted tubule within the nephron and is known for its high tendency to metastasize and an unfavorable prognosis compared with other non-ccRCC types, such as papillary and chromophobe RCCs (3). Frequent genetic abnormalities observed in ccRCC include loss of heterozygosity, hypermethylation, mutations, and deletions in the 3p chromosomal region. These genetic changes on chromosome 3p lead to inactivation of the von Hippel–Lindau (VHL) gene, which in turn reduces the production of the VHL protein (pVHL) (3–6). Compared with non-malignant adjacent kidney cortex and non-ccRCC types, ccRCC exhibits markedly reduced levels of pVHL (7, 8). These mutations impair pVHL function and facilitate the degradation of hypoxia-inducible factors (HIFs) under normoxic conditions. VHL encodes a protein component of the E3 ubiquitin ligase complex that targets the HIF-α subunits for degradation (9–11). As a result of pVHL dysfunction, the accumulation of HIFs promotes angiogenesis and tumor proliferation. Therefore, the pVHL protein status plays a crucial role in ccRCC pathogenesis (12).

HIFs are transcription factors that activate genes associated with various processes in response to hypoxia, including angiogenesis, metabolism, and cell survival. Under normoxic conditions, HIF-α subunits undergo hydroxylation, facilitating their recognition and targeting by VHL for proteasomal degradation (9). Nevertheless, this hydroxylation process is suppressed in low-oxygen environments, leading to stabilization and activation of HIF-α. Once activated, HIF-α translocates to the cell nucleus and triggers specific target genes. VHL-mediated control of HIF-α subunits plays a crucial role in the cellular adaptation to oxygen levels.

The HIF-α family comprises three unstable subunits: HIF-1α, HIF-2α, and HIF-3α, which are encoded by HIF1A, EPAS1, and HIF3A, respectively (13). Although they share similar protein structures and amino acid sequences, HIF-1α and HIF-2α have distinct functions (14). Both are implicated in the development, spread, and progression of renal cell carcinoma (RCC) (14). In contrast, the role of HIF-3α has not yet been fully elucidated, and it exhibits low amino acid sequence similarity with HIF-1α and HIF-2α (15). HIF-3α undergoes alternative splicing to produce various isoforms (16). Notably, the HIF-3α4 splice variant has a dominant-negative effect on the hypoxic response (17). Furthermore, HIF-3α functions as a positive transcriptional regulator of several downstream molecules, although its role in ontogeny remains unclear (17). HIF-α proteins are predominantly localized in the nucleus and show higher expression in ccRCC tissues than in non-ccRCC tumor tissues (18–20).

HIF-α activation results in E-cadherin suppression through the induction of transcriptional repressors such as Snail, SIP1, and TWIST (21). E-cadherin, a calcium-dependent cell adhesion molecule, is essential for preserving epithelial integrity and inhibiting tumor invasion. The reduction of E-cadherin is vital for initiating the epithelial-to-mesenchymal transition (EMT) and substantially increases cell motility and invasiveness (22). In VHL disease, VHL inactivation in precancerous lesions is strongly associated with a considerable decrease in E-cadherin expression (23). This suggests that E-cadherin loss may be an early event in the progression of ccRCC.

This study aimed to investigate the complex relationship between pVHL, HIFs, and E-cadherin in ccRCC to enhance our understanding of cellular responses to oxygen levels and their implications for cancer progression.

## Materials and methods

### Patient and clinical sample collection

A cohort of 181 patients underwent surgical intervention with radical or partial nephrectomy between 1988 and 2009 at the University Hospital Umeå, Sweden. All participants provided informed consent, and written informed consent was obtained from January 2000 to participate in this study. Participants were apprised that the studies encompassed survival information, laboratory values, measurements of tumor variables, and genetic alterations. The Institutional Review Board and Ethics Committee of Northern Sweden approved this study. Participants were informed of their right to withdraw from the study at any time for any reason.

Multiple tumor and kidney cortex tissue specimens were obtained from surgically excised tumor-bearing kidneys, formalin-fixed, and subjected to histological examination (24). RCC type was classified according to the Heidelberg classification (25), tumor stage was determined using the TNM classification (26), and nuclear grade was assessed using the Fuhrman grading system (27). The distribution of patient characteristics in relation to the RCC type is shown in Table 1. TNM stage groups I and II, and stages III and IV were aggregated. Similarly, Grades 1 and 2 and Grades 3 and 4 were aggregated. Patients were monitored using a structured follow-up program (Table 1).

**Table 1:** Distribution of patients’ characteristics in 150 patients with clear cell RCC subdivided into pVHL-low and pVHL-high levels.

Parameters		pVHL-low	pVHL-high	All ccRCC
		(*n* = 71)	(*n* = 72)	(*n* = 150)
**Age (years)**	**Mean**	64.7	65.6	65.8
	**Median (range)**	64 (32–85)	66 (38–84)	67 (34–87)
**Gender**	**Men**	30 (42%)	33 (54%)	43 (58%)
	**Women**	41 (58%)	39 (46%)	87 (63%)
**T-stage**	**T1**	32 (45%)	23 (32%)	48 (32%)
	**T2**	8 (11%)	16 (22%)	26 (17%)
	**T3**	11 (15.5%)	16 (22%)	31 (21%)
	**T4**	20 (28%)	17 (24%)	45 (30%)
**N-stage**	**No**	32 (45.1%)	23 (31.9%)	105 (70%)
	**N1**	39 (54.9%)	49 (68.1%)	45 (30%)
**Survival**	**Alive**	23 (33%)	21 (29%)	52 (33%)

pVHL: von Hippel–Lindau protein.

### Protein extraction and analysis

Proteins were extracted from clinical samples, as previously described (7, 28). In brief, to isolate protein from the clinical samples, the tissue was carefully chopped using a surgical knife. The samples were then placed on ice and shaken for 30 min, followed by centrifugation at 10,000 rpm for 10 min at 4°C. The supernatant containing the proteins was collected. Proteins were analyzed using bicinchoninic acid assay (BCA assay) (Thermo Fisher Scientific, Waltham, MA, USA) according to the manufacturer’s guidelines.

### Immunoblot

Protein samples (30 μg) were separated on NuPAGE Novex 10% or 12% gels (Life Technologies, Carlsbad, CA, USA) using an XCell SureLock™ Mini-Cell (Life Technologies) and then transferred to nitrocellulose membranes using a Trans-Blot Turbo transfer system (Bio-Rad Laboratories, Hercules, CA, USA). Membranes were subsequently blocked for 1 h at room temperature with either 5% BSA, 5% non-fat milk, or diluted Odyssey blocking buffer (Licor Biosciences, Lincoln, NE, USA) in Tris-buffered saline, depending on the antibodies used. Overnight incubation at 4°C with gentle agitation was performed using specific primary antibodies: E-cadherin (AB231303 Abcam, Cambridge, UK; 1: 1000), VHL (NB100-485, Novus Biologicals), and β-actin (A5316, Sigma-Aldrich, St. Louis, MO, USA). Secondary antibodies – IRDye^®^ 800CW Goat Anti-Rabbit (LI-COR #926-32211, LI-COR Biosciences) or IRDye^®^ 680CW Goat Anti-Mouse (LI-COR #925-68070, LI-COR Biosciences) – were used to detect the primary antibodies. An Odyssey CLx infrared imaging system (Licor Biosciences) was used for membrane visualization, while Image Studio System™ software version 3.1 (Licor Biosciences) was used for densitometry analysis. The relative density values for all proteins were determined by normalizing to the housekeeping protein, β-actin. Previous studies have shown that β-actin can be used for normalization in E-cadherin Western blot analysis of ccRCC, provided its limitations are recognized and data interpretation remains contextual (29). The methods and results obtained from our previous study on pVHL (7) were utilized in the present comparative study.

### Tissue microarray construction

Four representative tumors and two kidney cortex cores measuring 0.6 mm in diameter were placed in a newly prepared recipient paraffin block from previously formalin-fixed and paraffin-embedded tissue blocks. The tissue microarray (TMA) blocks were sliced into 4 μm sections and treated according to standard procedures, including deparaffinization and rehydration. A representative slice of each TMA block was stained with hematoxylin and eosin. The stained TMA sections were reviewed and confirmed by a clinical pathologist.

### Immunohistochemical staining

TMA sections were subjected to antigen retrieval using citrate buffer at pH 6, followed by 20-min blocking of endogenous peroxidase with 200 mL of methanol containing 3 mL of 40% H_2_O_2_. The sections were incubated with primary antibodies at the following dilutions: HIF-1α (NB100-132; Novus Biologicals, Cambridge, UK; 1:200), HIF-2α (NB100-134; Novus Biologicals; 1:150), and HIF-3α (ab10134; Abcam, Cambridge, UK; 1:200). The secondary antibody used was EnVision+ Dual-link Single Reagent (HRP, rabbit/mouse; Agilent, CA, USA). Visualization was achieved using diaminobenzidine/H_2_O_2_, and the sections were counterstained with hematoxylin. Immunohistochemistry (IHC) was performed on 150 ccRCC and 31 non-ccRCC samples. Owing to core loss during IHC, analyses were performed on 149, 149, and 148 ccRCCs for HIF-1α, HIF-2α, and HIF-3α, respectively.

### Scoring of protein expression in the cytoplasm and nucleolus

A Panoramic 250 scanner (3DHistech, Budapest, Hungary) was used to digitally capture IHC-stained TMA slides at a magnification of 40×. We used QuPath version 0.2.0-m429, an open-source image analysis software developed by the Centre for Cancer Research & Cell Biology at the University of Edinburgh, to organize disordered IHC-stained TMAs. During the evaluation process, all cores were assessed, and those deemed invalid (with less than 10% tumor content or containing artefacts) were manually removed.

To quantify TMAs, we implemented a straightforward automated semi-assisted approach using QuPath. After several processing steps and validations, we established an optimal threshold for identifying positive cells for each marker. Staining vectors were automatically analyzed for each scanned TMA slide, enabling detection of the total tissue area, differentiation of tumor from non-tumor regions within each core, and automatic cell identification. Positive cells were identified using an optical density threshold set for each core, which was then applied to the entire array, following validation by an expert pathologist. The histochemical score (H-score) was used to measure the staining intensity. This score was calculated by adding the percentage of staining multiplied by the corresponding intensity, which served as an indicator of expression level. The methodology and H-score derived from IHC techniques were part of our earlier research (18). These data were employed in the current comparative study.

### Statistical analysis

Statistical analysis was performed using IBM SPSS Statistics 29.0. The Mann–Whitney U-test was used to assess the differences in variable levels between the two independent groups. Survival curves were constructed using Kaplan–Meier plots and analyzed using the log-rank test. Statistical significance was determined by a two-sided *P*-value less than 0.05 for all tests.

## Results

### Expression of pVHL, E-cadherin, and HIF-α in ccRCC and adjacent kidney cortex tissues, and their association with clinicopathological parameters

The levels of pVHL were significantly reduced in tumor samples (*n* = 143) compared with kidney cortex tissues (*n* = 35) (*P* = 0.012). Furthermore, no association was identified between pVHL levels and any clinicopathological parameters (7).

**Figure 1:**
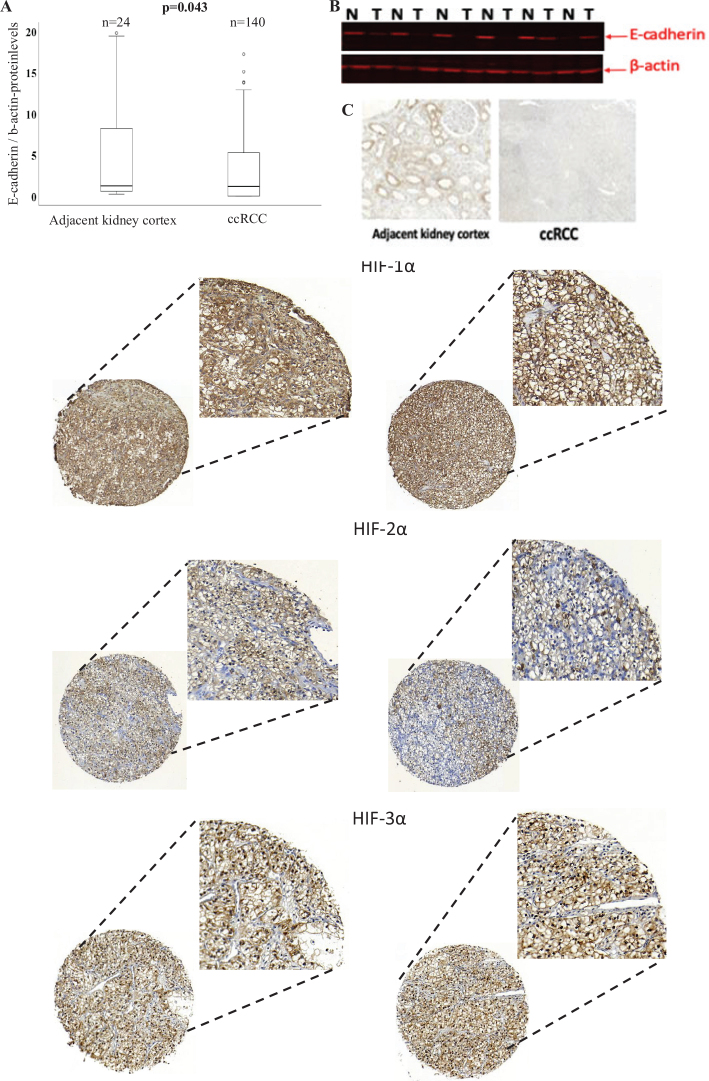
(a) Box plot illustrating the expression levels of E-cadherin protein in adjacent kidney cortex and ccRCC. (b) Representative immunoblots showing the protein expression of E-cadherin and the loading control β-actin in the adjacent kidney cortex (N) and ccRCC (T). (c) Representative stained tissue samples of ccRCC and adjacent kidney cortex after IHC staining with E-cadherin. (d) Representative stained tissue sections of ccRCC displaying nuclear HIF-1α, HIF-2α, and HIF-3α.

E-cadherin expression levels were lower in ccRCC tissues than in kidney cortex tissues (*P* = 0.043) (Figure 1a–c). No correlation was observed between E-cadherin expression and age or sex (data not shown). Additionally, E-cadherin levels were lower in advanced-stage and larger tumors compared with early-stage and smaller tumors (Table 2).

**Table 2:** Relation of E-cadherin levels to categorized clinicopathological parameters in 142 patients with ccRCC.

Parameter	*n*	E-cadherin Median (IQR)	Mean	*P*
**Tumor grade**				
1–2	16	4.03 (0–17.21)	5.27	0.097
3–4	124	0.99 (0–19.38)	3.054	
**TNM stage**				
I–II	77	2.2 (0–19.38)	3.77	**0.049**
III–IV	63	0.4 (0–12.89)	2.72	
**Tumor diameter**				
**≤ 70 mm**	69	2.2 (0–19.38)	4.13	**0.032**
**≥ 70 mm**	71	0.45 (0–11.70)	2.5	

ccRCC: clear cell renal cell carcinoma; n: number of patients; tumor grade: Fuhrman grade classification; TNM stage groups; IQR: interquartile range.

Groups were compared using the Mann–Whitney U-test (significant at *P* < 0.05). Significant *P*-values are given in bold.

The protein levels of HIF-1α (*P* = 0.03), HIF-2α (*P* = 0.03), and HIF-3α (*P* = 0.028) were higher in the nucleus than in the cytoplasm (Figure 1d) (18). The nuclear expression of HIF-1α was significantly lower in TNM stage I (*n* = 47) than in stage IV (*n* = 43; *P* = 0.043). In contrast, nuclear HIF-2α expression was significantly lower in smaller tumors (*n* = 66) than in larger tumors (*n* = 83; *P* = 0.035). No significant correlation was found between the nuclear expression of HIF-3α and clinicopathological parameters (18).

### Relation between pVHL status versus E-cadherin and HIF-1α, HIF-2α, and HIF-3α protein expression levels

**Figure 2:**
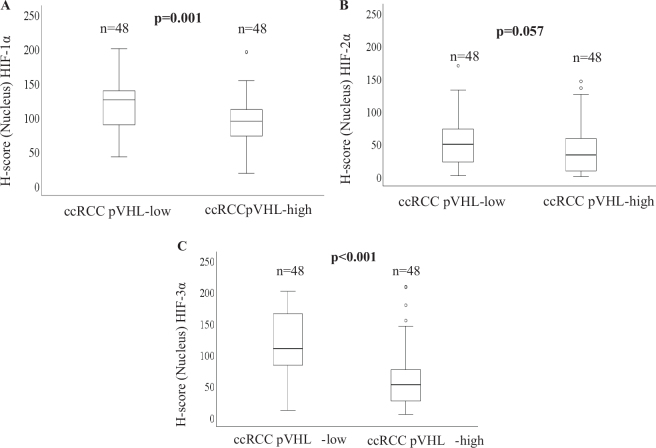
Box plots comparing the expression levels of (a) HIF-1α in pVHL-low ccRCC and pVHL-high ccRCC, (b) HIF-2α in pVHL-low ccRCC and pVHL-high ccRCC, and (c) HIF-3α in pVHL-low ccRCC and pVHL-high ccRCC.

Based on the median pVHL level, patients with ccRCC were divided into two subgroups: pVHL-low ccRCC (*n* = 71) and pVHL-high ccRCC (*n* = 72) (7). Patients with pVHL-low ccRCC exhibited significantly higher levels of HIF-1α (*P* = 0.001) and HIF-3α (*P* < 0.001) than those with pVHL-high ccRCC, whereas there was no significant difference in HIF-2α expression (*P* = 0.057) between pVHL-low and pVHL-high ccRCCs (Figure 2). E-cadherin expression was lower in advanced stages and larger tumors in pVHL-high ccRCCs. In contrast, there was no association between E-cadherin expression and clinicopathological variables in pVHL-low ccRCCs (Table 3).

**Table 3:** Relation of E-cadherin protein levels to clinicopathological parameters in 128 patients with clear cell RCC subdivided into pVHL-low and pVHL-high.

Parameter	*n*	pVHL-low ccRCC				pVHL-high ccRCC		
		E-cadherin				E-cadherin		
		Median (IQR)	Mean	*P*	*n*	Median (IQR)	Mean	*P*
**Tumor grade**								
1–2	9	4.22 (0–17.21)	5.39	0.758	4	1.62 (0–12.89)	4.03	0.893
3–4	57	0.99 (0–15.10)	2.96		56	1.2 (0–19.38)	3.26	
**TNM stage**								
I–II	38	1.145 (0–17.21)	3.45	0.793	31	2.79 (0–19.38)	4.16	**0.022**
III–IV	28	1.11 (0–9.48)	3.068		29	2.4 (0–12.89)	2.4	
**Tumor diameter**								
**≤ 70 mm**	38	1.035 (0–17.21)	3.35	0.758	25	4.32 (0–19.38)	5.34	**0.002**
**≥ 70 mm**	28	2.18 (0–9.58)	3.2		35	0.38 (0–11.74)	1.86	

*pVHL*: von Hippel–Lindau protein; ccRCC: clear cell renal cell carcinoma; n: number of patients; tumor grade: Fuhrman grade classification; TNM stage: TNM stage groups; IQR: interquartile range.

Groups were compared using the Mann–Whitney U-test (significant at *P* < 0.05). Significant *P*-values are given in bold.

E-cadherin protein expression levels were inversely correlated with nuclear levels of HIF-1α (*P* = 0.043) but showed no correlation with HIF-2α (*P* = 0.247) or HIF-3α (*P* = 0.467), as shown in Table 4. In pVHL-high ccRCC, E-cadherin expression was inversely related with HIF-1α expression (*P* = 0.009), whereas in pVHL-low ccRCC, E-cadherin expression was negatively correlated with HIF-2α expression (*P* = 0.027; Table 4). No correlation was observed between E-cadherin and HIF-3α expression in either pVHL-high or pVHL-low ccRCC (Table 4).

**Table 4:** Association between E-cadherin and nuclear HIF-α expression levels in ccRCC, subdivided into pVHL-low and pVHL-high patients.

Parameter	Hif-1α (nuclear)	Hif-2α (nuclear)	Hif-3α (nuclear)
**ccRCC**			
**E-cadherin**	*P* = 0.043*	*P* = 0.247	*P* = 0.467
	*r* = −0.198	*r* = −0.114	*r* = −0.073
	*n* = 105	*n* = 105	*n* = 103
**pVHL-low ccRCC**			
**E-cadherin**	*P* = 0.961	*P* = 0.027*	*P* = 0.875
	*r* = −0.007	*r* = −0.323	*r* = −0.024
	*n* = 47	*n* = 47	*n* = 47
**pVHL-high ccRCC**			
**E-cadherin**	*P* = 0.009*	*P* = 0.746	*P* = 0.744
	*r* = −0.380	*r* = −0.049	*r* = −0.050
	*n* = 47	*n* = 47	*n* = 45

pVHL: von Hippel–Lindau protein; ccRCC: clear cell renal cell carcinoma.

* Spearman’s correlation analyses (significant at *P* < 0.05).

### Association between pVHL status, E-cadherin, and HIF-1α, HIF-2α, and HIF-3α protein expression levels and cancer specific survival

**Figure 3:**
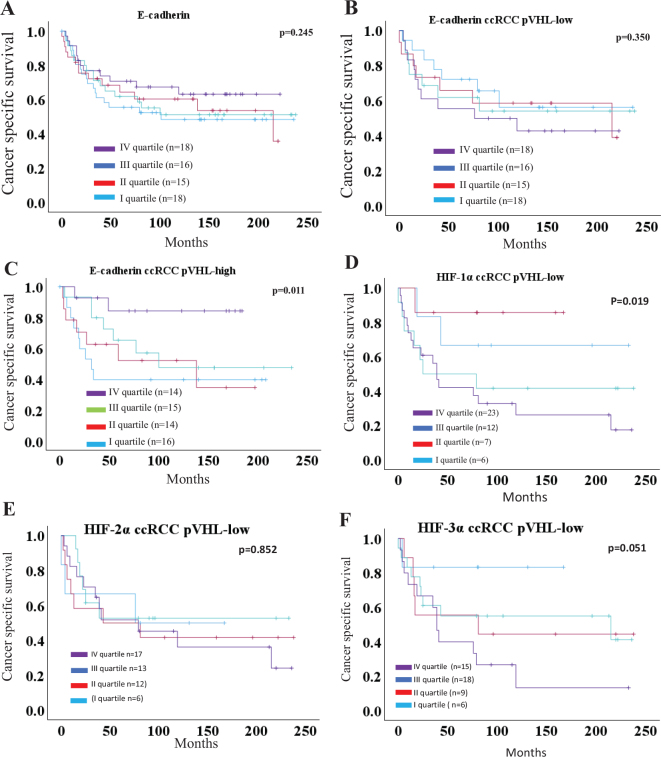
Kaplan-Meier plots displaying cancer-specific survival curves for ccRCC: (a) E-cadherin, (b) E-cadherin pVHL-low ccRCC, (c) E-cadherin pVHL-high ccRCC, (d) nuclear HIF-1α pVHL-low ccRCC, (e) HIF-2α pVHL-low ccRCC, (f) HIF-3α pVHL-low ccRCC.

No correlation was identified between E-cadherin expression and CSS (Figure 3a). However, in patients with pVHL-high ccRCC, higher E-cadherin levels were associated with CSS (*P* = 0.011) (Figure 3b). Conversely, there was no association between E-cadherin expression and CSS in patients with pVHL-low ccRCC (*P* = 0.350) (Figure 3c).

Patients with high nuclear HIF-1α and HIF-3α expression levels showed significantly reduced CSS (*P* = 0.002 and *P* = 0.019, respectively), whereas HIF-2α expression levels (*P* = 0.12) were not associated with CSS (7). In patients with pVHL-low RCC, a significant survival benefit was observed in tumors with lower nuclear HIF-1α levels (Figure 3d). However, neither HIF-2α (*P* = 0.852) nor HIF-3α (*P* = 0.051) was significantly associated with survival (Figure 3e–f). No difference in survival was observed between the subgroups of patients with pVHL-high ccRCC (data not shown).

## Discussion

VHL plays a pivotal role in the regulation of E-cadherin expression, which is crucial for the ontogeny and progression of ccRCC. VHL facilitates oxygen-dependent degradation of HIF-α subunits and is a key factor in this regulation. VHL inactivation results in HIF accumulation and E-cadherin inhibition in ccRCC cells. Our study confirmed an inverse relationship between the levels of E-cadherin, HIF-1α, and HIF-2α in ccRCC.

Our study showed significantly reduced pVHL expression in ccRCC compared with adjacent non-tumor tissues and non-ccRCC tumors (7), which aligns with the results of previous studies (8, 14). Notably, no significant correlation was observed between clinicopathological factors and pVHL expression in ccRCC and pVHL-low or pVHL-high ccRCC (7). Our ccRCC cohort lacked VHL genomic and epigenomic data, which would have strengthened our understanding of the underlying mechanism. However, the absence of this information did not restrict our ability to interpret the key biological changes within this cohort, such as HIF activation and E-cadherin downregulation. These molecular alterations can occur through various converging pathways (30, 31), thereby remaining relevant to the biology of ccRCC, even in the absence of specific data on VHL aberrations. These results indicate that VHL mutations or deletions alone are insufficient to drive ccRCC progression (31). Inactivation of the VHL gene and the subsequent increase in HIF, which characterize most sporadic ccRCCs, stimulate various growth factors (32). Consequently, the VHL–HIF pathway is intricately linked and plays a role in the development of ccRCC through PI3K, Wnt, and several other signalling cascades (30, 31). In ccRCC, the expression levels of HIF-1α, HIF-2α, and HIF-3α are significantly higher in the nucleus than in the cytoplasm. Furthermore, the expression of nuclear HIF-1α is strongly correlated with the levels of both nuclear HIF-2α and HIF-3α, whereas HIF-2α is associated only with HIF-1α (18). The study also revealed a significant correlation between CSS and the nuclear expression of HIF-1α and HIF-3α, indicating that these proteins play pivotal roles in angiogenesis and proliferation in ccRCC (18). Various HIF-α subunits (HIF-1α, HIF-2α, and HIF-3α) use distinct nuclear localization signals (NLS) to enter the nucleus. Although HIF-1α and HIF-2α utilize a bipartite NLS in their C-terminal domains, HIF-3α features two redundant NLS motifs in its unique C-terminal region (33, 34). The present study showed that nuclear HIF-1α expression was significantly higher in pVHL-low ccRCC than in pVHL-high ccRCC. In contrast, the nuclear expression of HIF-2α was not significantly different between pVHL-low and pVHL-high ccRCC. These disparities among HIF-α subunits and pVHL status likely contribute to their unique roles in the hypoxia response and gene regulation. Although HIF-1α and HIF-2α employ similar nuclear import mechanisms, they demonstrate distinct differences in the tissue distribution, temporal dynamics, and regulatory mechanisms governing their nuclear localization (33–38). Our study showed that nuclear localization of HIF-3α was significantly higher in pVHL-low than in pVHL-high ccRCC. Although previous studies have not specifically reported the role of VHL in HIF-3α, it is plausible to hypothesize that VHL plays a role in regulating HIF-3α, similar to its regulatory function in HIF-1α and HIF-2α. Further research is required to elucidate this hypothesis and clarify the underlying mechanisms.

In this study, we observed a significantly lower E-cadherin expression in ccRCC than in the kidney cortex. These findings are consistent with previous studies (39, 40). Similar to earlier studies, low E-cadherin expression correlated with larger tumor sizes and advanced tumor stages (40, 41). Moreover, among the patients with pVHL-high ccRCC, we identified a significant link between E-cadherin expression and CSS, unlike in the pVHL-low subgroup. These results suggest that reduced E-cadherin expression is linked to aggressive behavior in tumors with high pVHL levels.

The HIF pathway primarily mediates VHL regulation of E-cadherin (23). We observed a negative correlation between E-cadherin and nuclear HIF-1α, HIF-2α, and HIF-3α expression levels. These findings are corroborated by previous studies demonstrating the crucial role of HIF-1α in E-cadherin suppression, as it indirectly enhances the expression of transcriptional repressors, including TCF3, ZFHX1A, and ZFHX1B (42). In contrast, HIF-2α exerts an indirect inhibitory effect on E-cadherin transcription by enhancing transcriptional repressors such as Snail and SIP1 (21). Although the interaction between HIF-3α and pVHL is novel, the precise mechanism by which HIF-3α suppresses E-cadherin is unknown and possibly involves an indirect complex mechanism (43).

In conclusion, loss of the VHL gene in ccRCC leads to constitutive HIF-α activation and E-cadherin repression. These interactions play pivotal roles in cancer progression by enhancing angiogenesis, invasion, metastasis, metabolic reprogramming, and resistance to various therapies. However, the exact underlying mechanisms require further investigation. Therapeutic implications suggest potential treatment targets for cancer, including anti-angiogenic therapies, HIF inhibitors, and strategies to maintain E-cadherin expression.

## Data sharing statement

The datasets relevant to the current study are submitted with the article.
